# Macromolecular nanoparticles to attenuate both reactive oxygen species and inflammatory damage for treating Alzheimer's disease

**DOI:** 10.1002/btm2.10459

**Published:** 2022-11-29

**Authors:** Bosong Zhang, Yufang Zhao, Kai Guo, Hui Tian, Cao Wang, Ruiqi Wang, Yue Chen, Xiongbiao Chen, Hongxia Zheng, Bingxin Gao, Jieyi Shen, Weiming Tian

**Affiliations:** ^1^ School of Life Science and Technology Harbin Institute of Technology Harbin China; ^2^ Laboratory for Space Environment and Physical Sciences Harbin Institute of Technology Harbin China; ^3^ Department of Mechanical Engineering, College of Engineering University of Saskatchewan Saskatoon Canada; ^4^ Division of Biomedical Engineering, College of Engineering University of Saskatchewan Saskatoon Canada; ^5^ Shandong Junxiu Biotechnology Co, Ltd Yantai China

**Keywords:** Alzheimer's disease, drug delivery, hyaluronic acid, polyphenols, reactive oxygen species

## Abstract

Prevention and early intervention are the current focus of treatment for Alzheimer's disease (AD). An increase in reactive oxygen species (ROS) is a feature of the early stages of AD, thus suggesting that the removal of excess ROS can be a viable method of improving AD. Natural polyphenols are able to scavenge ROS and thus promising for treating AD. However, some issues need to be addressed. Among them, important are that most polyphenols are hydrophobic, have low bioavailability in the body, are easily degraded, and that single polyphenols have insufficient antioxidant capacity. In this study, we employed two polyphenols, resveratrol (RES) and oligomeric proanthocyanidin (OPC), and creatively grafted them with hyaluronic acid (HA) to form nanoparticles to address the aforementioned issues. Meanwhile, we strategically grafted the nanoparticles with the B6 peptide, enabling the nanoparticles to cross the blood–brain barrier (BBB) and enter the brain for AD treatment. Our results illustrate that B6‐RES‐OPC‐HA nanoparticles can significantly scavenge ROS, reduce brain inflammation, and improve learning and memory ability in AD mice. B6‐RES‐OPC‐HA nanoparticles have the potential to prevent and alleviate early AD.

## 1 INTRODUCTION

Alzheimer's disease (AD) patients have significantly elevated levels of reactive oxygen species (ROS) in the brain and experience oxidative stress.^1^, ^2^ In AD, the abnormal mitochondrial metabolism produces ROS represented by hydroxyl radicals (HO•) and superoxide radicals (O2•).^3^ Studies have shown that ROS triggers inflammatory signaling activation and apoptosis, alters the function of amyloid β (Aβ) and τ proteins, and impairs cognitive performance in mice.^1^, ^4^ Acetylcholinesterase inhibitors (AChEIs) and *N*‐methyl‐d‐aspartate receptor (NMDAr) antagonists are commonly used in clinical practice.^5^, ^6^ Hormones, antidepressants, and atypical drugs have also been used to treat AD. However, all have failed to reverse the disease.^7^ It could be more effective to treat the disease before the onset of apparent symptoms.^8^ In the early stages of AD, ROS and inflammation in the nervous system are already significantly elevated.^9^ This suggests that preventing excessive ROS accumulation could be the key to AD prevention and treatment.

Polyphenols, commonly found in the daily diet, have been illustrated with ROS‐scavenging ability.^10^, ^11^ Resveratrol (RES), one of the polyphenols, can scavenge ROS, thus demonstrating its anti‐AD effect. Indeed, studies were recently performed to alleviate AD symptoms by using the antioxidant activity of RES, which was strategically incorporated in nanoparticles.^12^, ^13^ Notably, the antioxidant power of polyphenols is derived from the phenolic hydroxyl groups, which have varying effects on radical scavenging, depending on their substituents.^14^, ^15^ The antioxidant capacity of a single polyphenol is limited by the type and number of phenolic hydroxyl groups. Another polyphenol, oligomeric proanthocyanidin (OPC), has a strongly antioxidant capacity, suggesting its potential as a therapeutic agent for AD.^16^ Meanwhile, it is noted that polyphenols for oral administration have low concentrations in plasma, and their half‐life is very short in vivo.^17^, ^18^, ^19^ Plus, excessive concentrations of polyphenols can be toxic. As a result, protecting polyphenols in vivo and delivery to the nervous system have been challenging and remain to be addressed. Our previous studies have demonstrated that hyaluronic acid (HA) has good neuroinflammation resistance,^20^ as well as suitable biocompatibility in many biomedical applications.^21^, ^22^, ^23^, ^24^, ^25^, ^26^, ^27^ Studies have shown that mitochondrial abnormalities are the leading cause of ROS accumulation in the nervous system.^28^ Further, our recent studies found that HA can reduce ROS and restore neural damage by protecting the mitochondria.^29^ In addition, one significant challenge for nanoparticle delivery of polyphenols for AD treatments is the issue associated with the blood–brain barrier (BBB),^30^ which is formed as the interface between blood and the brain to protect the brain by restricting the passage or crossing of solutes, such as antibodies and drugs. The B6 peptide utilizes the transferrin in the BBB as a targeting site to efficiently assist nanoparticles entering the central nervous system.^31^, ^32^, ^33^ Therefore, polyphenols and HA, along with the B6 peptide, are the rationale for developing or constructing nanoparticles, which can cross the BBB, further scavenge the central nervous ROS, and reduce AD damage.

**SCHEME 1:**
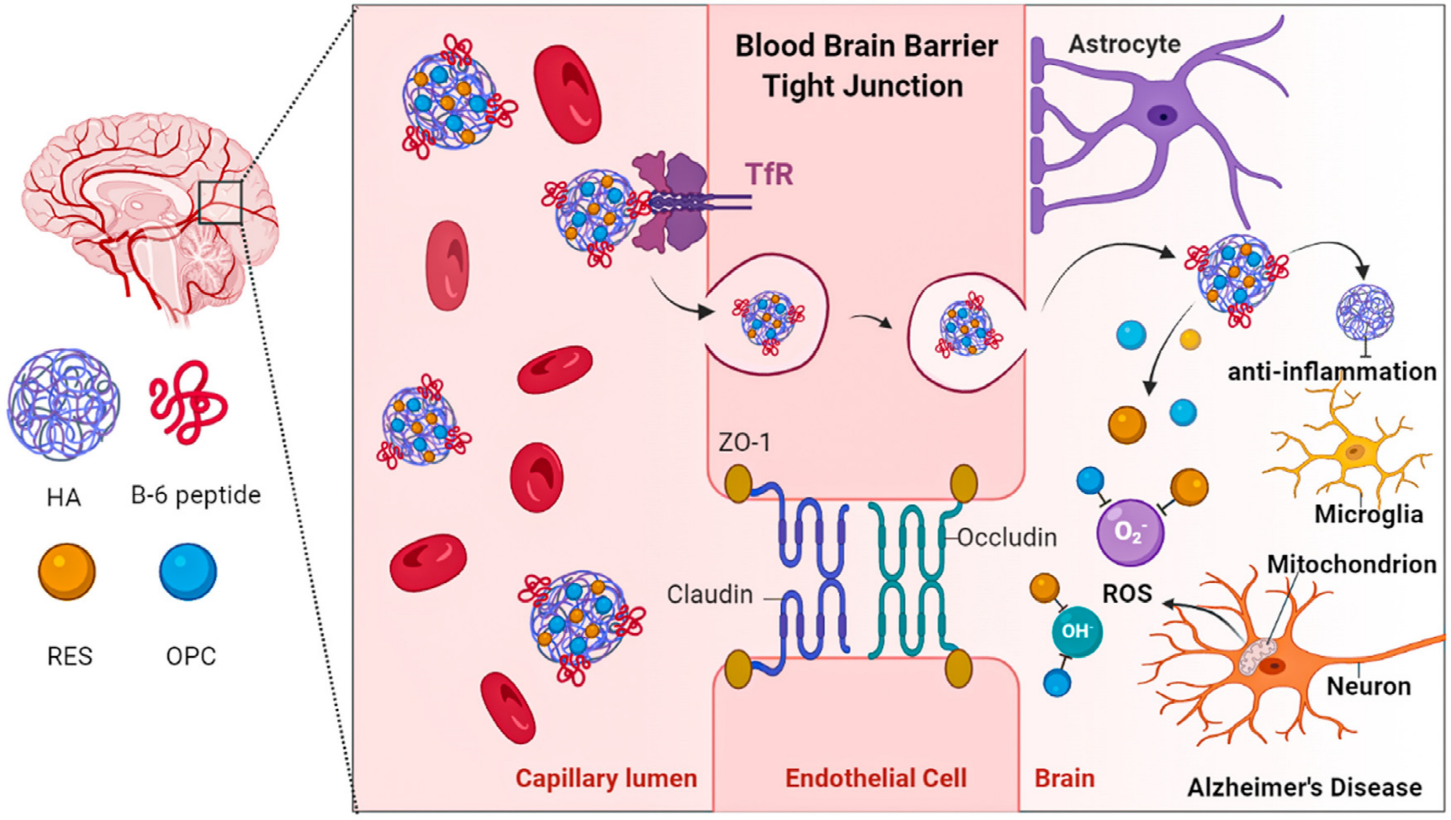
Macromolecular nanoparticles were designed based on hyaluronic (HA), grafted with resveratrol (RES), oligomeric proanthocyanidins (OPC) and B6 peptides. The nanoparticles protect the antioxidant capacity of the polyphenols in vivo and exploit the targeting of transferrin (TfR) of B6 peptides to cross the blood–brain barrier (BBB). Alzheimer's disease (AD) causes the accumulation of reactive oxygen species (ROS) in the brain and inflammation. Nanoparticles harness the anti‐inflammatory effects of HA and the scavenging effect of RES and OPC on ROS to achieve AD treatment

As inspired, in the present study, we used HA as the core material, grafted with two polyphenols (RES and OPC) and B6 peptides, to construct nanoparticles and further investigated their potential for ROS scavenging and neuroinflammation inhibition, as well as their ability to cross the BBB. Our innovative design of B6‐RES‐OPC‐HA nanoparticles (B6‐RES‐OPC‐HA NPs) and their strategical delivery into the brain allow for more efficient ROS clearance and neuroinflammation inhibition, thus effectively treating AD in early stages (Scheme 1).

## 2 RESULTS

### 2.1 Construction and characterization of B6‐RES‐OPC‐HA NPs

**FIGURE 1:**
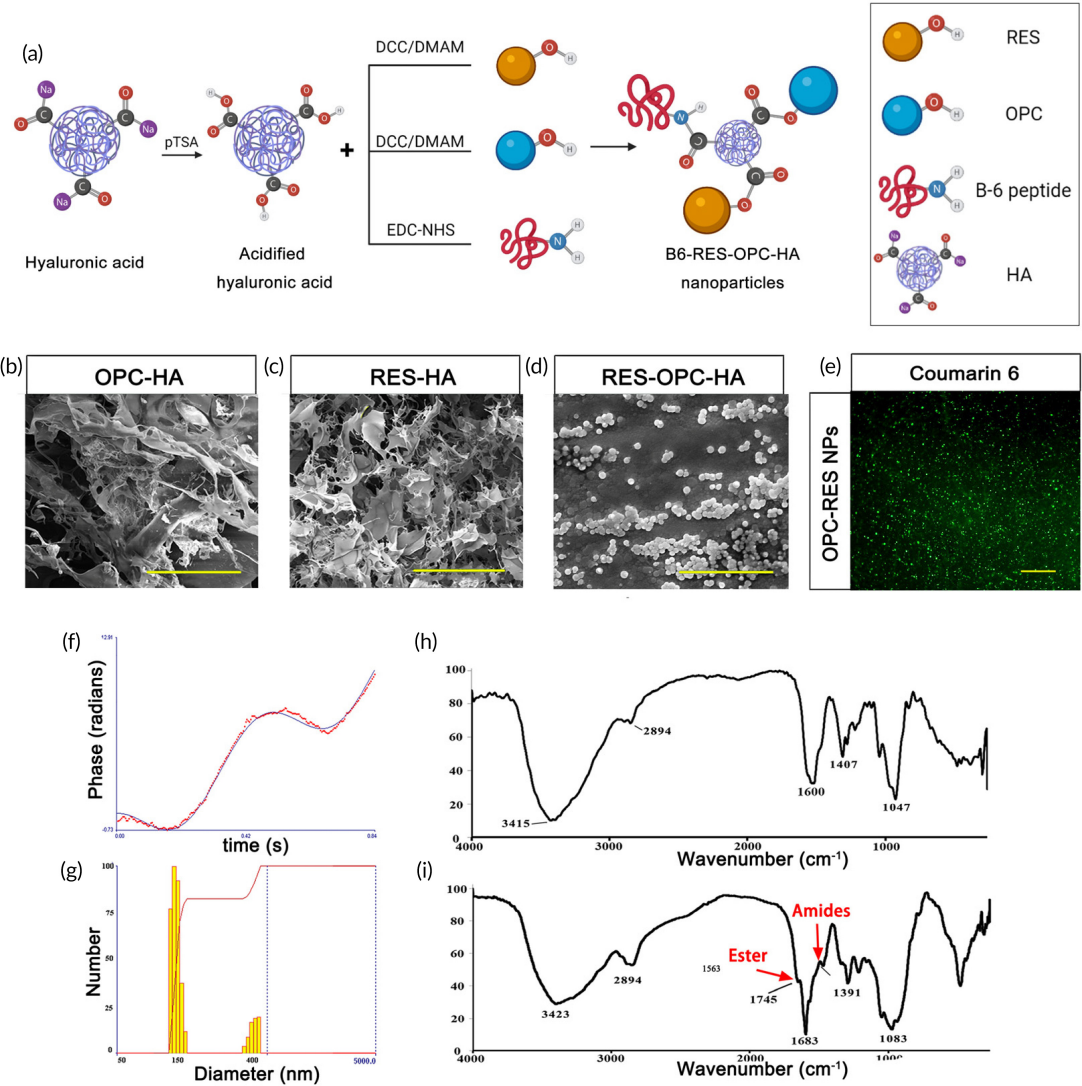
Synthesis and characterization of nanoparticles. (a) Acidification of hyaluronic (HA) to obtain activated carboxyl groups. The hydroxyl group in resveratrol (RES) and proanthocyanidins (OPC) forms an ester bond with the carboxyl group in HA, and the amino group in the peptide forms a peptide bond with HA. (b) Microstructure of OPC‐grafted HA, Scale bars = 100 μm. (c) Microstructure of RES‐grafted HA, Scale bars = 200 μm. (d) RES, OPC‐grafted HA simultaneously to form a dispersion sphere structure, Scale bars = 2 μm. (e) Confocal microscopy characterization of fluorescently labeled RES‐OPC‐HA nanoparticles, Scale bars = 100 μm. (f) Nanoparticle potential analysis. (g) Nanoparticle size analysis. (h) HA infrared spectra. (i) Infrared spectra of B6‐HA‐RES‐OPC NPs

The hydrophobicity of polyphenols is the key reason for their low bioavailability. Polyphenols' hydrophobicity is generally considered benzene ring related.^34^ Using HA crosslinked with polyphenols to construct nanoparticles could alleviate this problem (Figure 1a and Figure S1a,b).^35^ We examined the influence of crosslinking ratio by experiments and found that if the RES to HA molar ratio was 1:1, hydrophilic gels were obtained because the RES monomer was of the only two benzene‐ring structures and insufficiently hydrophobic (Figure 1b). In contrast, OPC monomer had four benzene rings, and hydrophobic flocculent precipitates were obtained at the OPC to HA molar ratio of 1:1 (Figure 1c). Finally, we found that using 11 W molecular weight HA with the HA:RES:OPC molar ratio of 2:2:1 could form stable and dispersed nanoparticles in the aqueous phase for a long time (Figure 1d). At the same time, the amino group in the B‐6 peptide was used to crosslink with the carboxyl group to form a peptide bond to assist the nanoparticles in entering the BBB (Figure 1a). The dispersed nanoparticle particles were observed by fluorescence microscopy when labeled with coumarin‐6 (Figure 1e, Figure S2a). Using zeta potential analysis, we measured a nanoparticle potential of −24.91 ± 1.88 mV and an average particle size of 279.8 ± 6 nm, consistent with the electron microscopic observations (Figure 1f,g). The lower potential and smaller particle size contribute to the stable presence of nanoparticles in blood circulation and across the BBB. Furthermore, for the constructed nanoparticles, we identified the characteristic peaks of ester bonds at 1745 cm^−1^ and amide bonds at 1391 cm^−1^ using infrared spectroscopy (Figure 1h,i). Also, we have verified the successful grafting of both polyphenols using nuclear magnetic resonance (Figure S2b) and confirmed that the polyphenols and B6 peptides were successfully grafted into HA and crosslinked to form stable nanoparticles.

### 2.2 HA‐RES‐OPC nanoparticles significantly scavenged ROS and reduced inflammation in vitro

**FIGURE 2:**
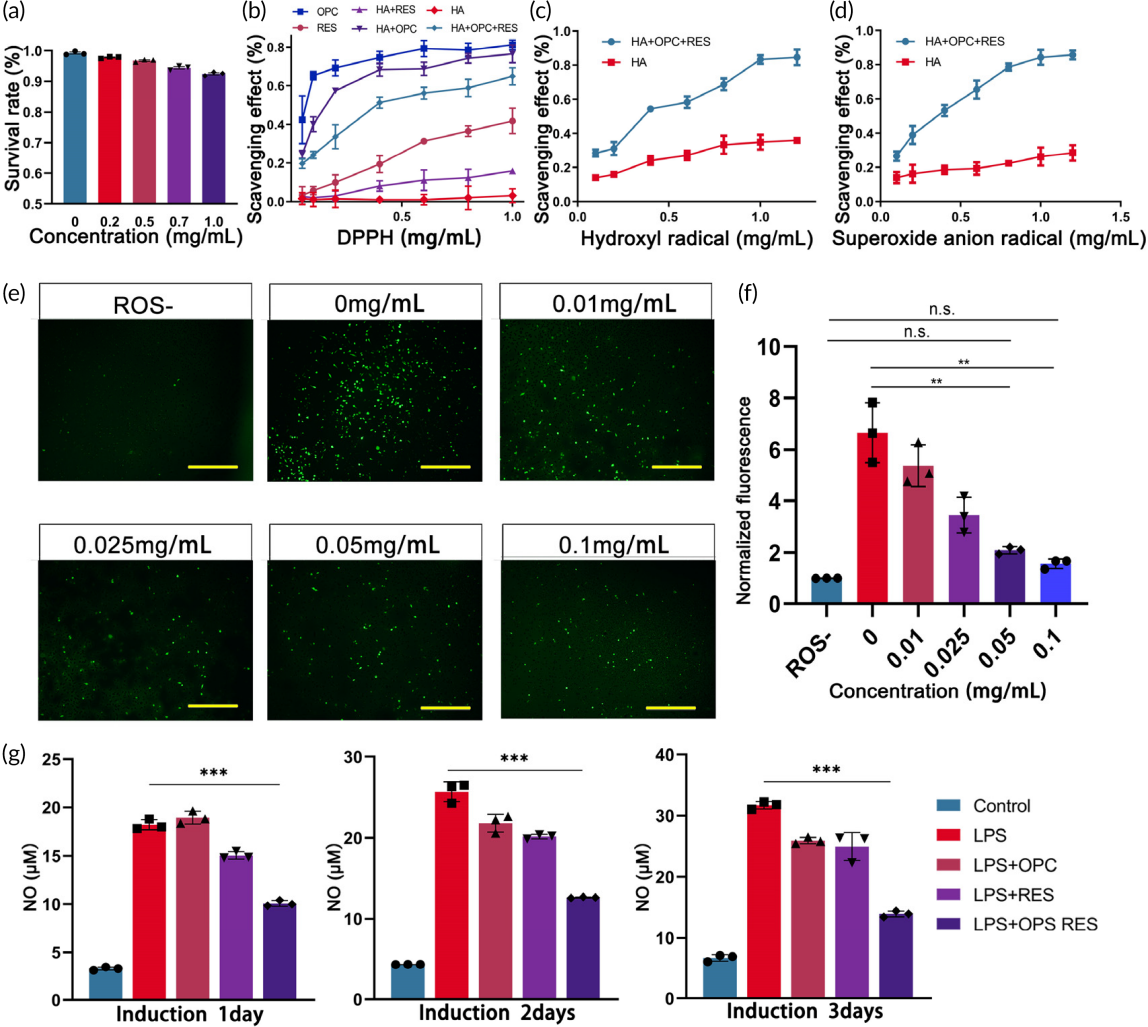
Nanoparticle antioxidant capacity detection. (a) MTT test for different concentrations of HA‐RES‐OPC NPs (*n* = 3). (b) DPPH to verify several nanoparticles' free‐radical scavenging ability. (c) The ability of nanoparticles to scavenge hydroxyl radicals. (d) The ability of nanoparticles to scavenge superoxide radicals. (e) Top left is the inflammation‐inactivated control, while the rest are treated with different concentrations of HA‐RES‐OPC nanoparticles; the cells with inflammation activation are marked in green in the figures where the scale bars are 1 mm. (f) ROS fluorescence intensity (*n* = 3). (g) Nitric oxide inhibition by nanoparticles on microglia in an inflammatory state. **p < 0.01, ***p < 0.001 (*n* = 3)

OPC and RES have good antioxidant properties and anti‐inflammatory effects in their monomeric state. In this regard, MTT experiments showed that when SH5Y5Y cells were treated even with a high concentration of HA‐RES‐OPC nanoparticles (1 mg/ml) for 12 h, the cell survival rate still reached more than 90%, indicating that RES‐OPC‐HA NPs have good biocompatibility (Figure 2a). 2,2‐Diphenyl‐1‐picrylhydrazyl (DPPH) is a stable free radical and is often used to determine the antioxidant capacity of compounds.^36^ In our study, the free‐radical scavenging ability of HA‐RES, HA‐OPC, HA‐RES‐OPC nanoparticles and RES, OPC was tested using DPPH. The ability of the OPC monomer to scavenge free radicals was significantly higher than that of RES, each at the same concentration. This result is consistent with the structure of the two polyphenols. Using HA crosslinked with polyphenols, the ability of both polyphenols to scavenge free radicals was reduced. However, the HA‐RES‐OPC nanoparticles formed using two polyphenols showed approximately five times the free‐radical scavenging capacity compared to the HA‐RES compound, similar to the OPC capacity (Figure 2b).

Superoxide radicals are mainly produced and progressively metabolized to hydroxyl radicals during the mitochondrial energy supply of the brain.^37^ After the modification with two polyphenols, the HA‐RES‐OPC nanoparticles are about three times more capable of scavenging hydroxyl radicals and about four times more capable of scavenging superoxide radicals compared to HA (Figure 2c,d). Next, the nanoparticles were assayed for their resistance to oxidative stress. ROSUP was used to simulate oxidative stress, and the fluorescent probe DCFH‐DA to verify the protective effect of adding different concentrations of nanoparticles on SH‐SY5Y cells. For the case where cells were treated with nanoparticles at concentrations higher than 0.05 mg/ml, the nanoparticles had a significant protective effect and did not differ significantly from cells that had not been treated with oxidative stress. These results indicate that nanoparticles significantly protect against cellular oxidative stress (Figure 2e,f). Inflammation dominates several processes in developing neurological diseases, and inflammatory processes in the nervous system are often associated with microglia. Several recent studies have shown that microglia play an important role in neurodegenerative diseases by regulating inflammation.^38^, ^39^ In our study, microglia were used to verify whether nanoparticles could alleviate cellular inflammation. Lipopolysaccharide (LPS) can activate microglia by mimicking bacterial infection. During cellular inflammation, the nitric oxide (NO) level allows for a precise evaluation of inflammation.^40^ The inhibitory effect of nanoparticles on cellular inflammation was characterized by measuring the amount of NO in the cell culture medium after LPS stimulation. Our results showed that microglia underwent significant inflammation induced by LPS. During the 3‐day induction, HA‐RES‐OPC nanoparticles showed significant inhibition of inflammation, with much more improvement than HA‐RES and HA‐OPC nanoparticles. This suggests that our constructed nanoparticles can significantly reduce cellular inflammation and protect the nervous system during a prolonged process (Figure 2g).

### 2.3 Grafted B6 peptide enhances the ability of nanoparticles to cross the BBB

**FIGURE 3:**
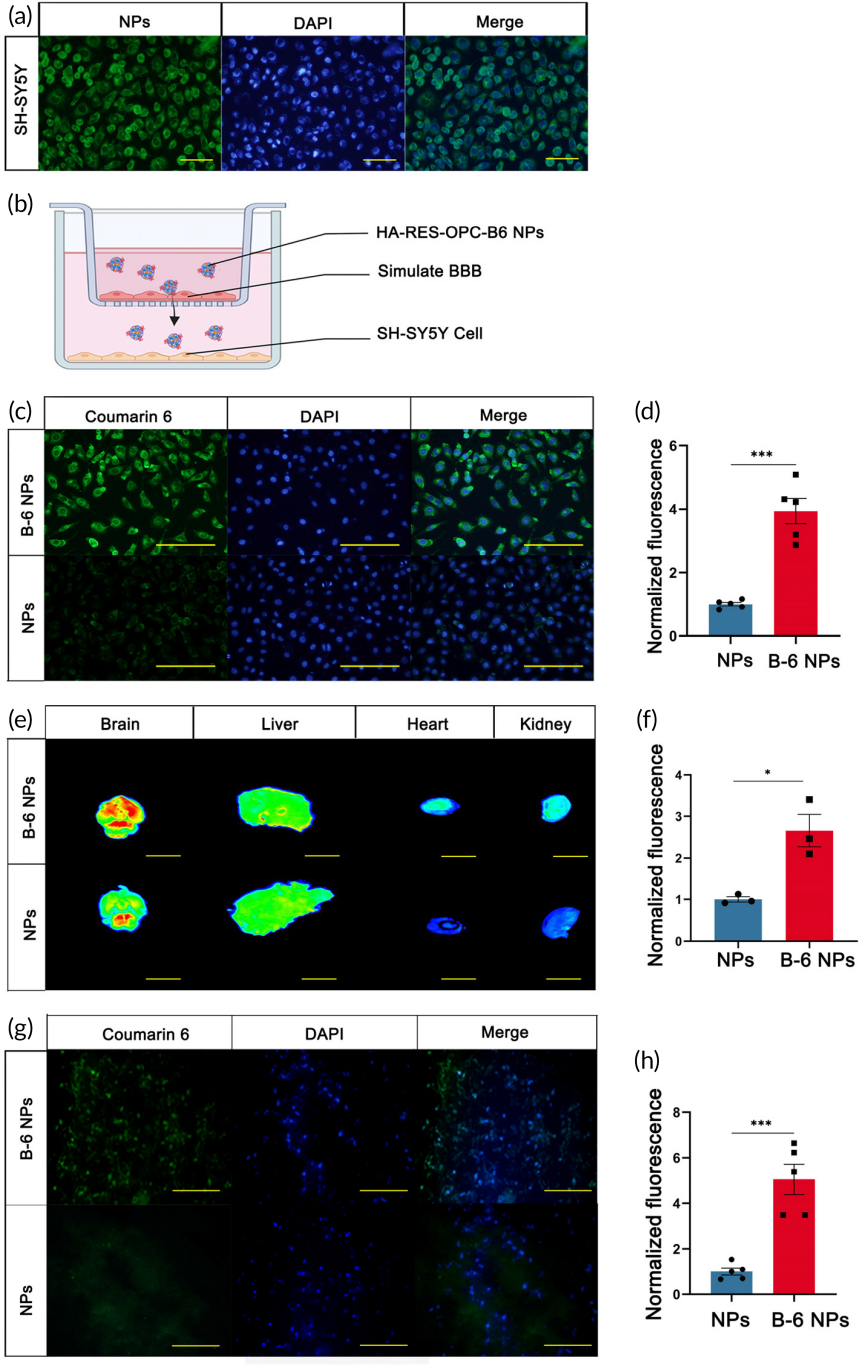
Validation of nanoparticle BBB crossing ability. (a) SH‐SY5Y cells treated with 10 μg/ml B6‐HA‐RES‐OPC NPs for 3 h, scale bars are 100 μm. (b) Validation of B6‐HA‐RES‐OPC NPs crossing ability using Transwell simulation of the blood–brain barrier (BBB). (c) Ability to cross the BBB after treatment of cells with two nanoparticles at a concentration of 10 μg/ml for 3 h, scale bars are 200 μm. (d) The ability of the nanoparticles to cross the BBB model is increased approximately fourfold after B6 grafting (*n* = 5). (e) Nanoparticle accumulation in brain, liver, heart, and kidney before and after grafting B6 peptide during nanoparticle treatment, scale bars are 5 mm. (f) Differences in the amount of nanoparticle deposition in the mouse brain after B6 grafting (*n* = 3). (g) Brain sections of mice treated with B6‐HA‐RES‐OPC NPs and HA‐RES‐OPC NPs, scale bars are 100 μm. (h) Ability of the nanoparticles to cross the BBB is increased approximately fourfold after grafting the B6 peptide. *p < 0.05, **p < 0.01, ***p < 0.001 (*n* = 5).

The BBB prevents nanoparticles from entering the cerebral nervous system from the blood circulation in the brain. The B6 peptide is a small molecule protein that targets transferrin at the BBB and enables nanoparticle transport across the membrane. In our study, we attached the B6 peptide to nanoparticles and verified the ability of nanoparticles to cross vascular endothelial cells. SH‐SY5Y cells were treated with the fluorescein‐labeled B6‐RES‐OPC‐HA NPs at a concentration of 10 μg/ml for 3 h, with the results illustrating that nanoparticles had the ability to enter cells (Figure 3a). We constructed an astrocyte and brain microvascular endothelial cells (BMEC) co‐culture system to mock BBB for nanoparticle characterization. For this, we mixed BMECs and C8‐D1A cells in the upper layer of the transwell and cultured SH‐SY5Y cells in the lower layer. The cells in the upper layer formed a dense network structure for subsequent experiments **(**Figure 3b). After adding fluorescein‐labeled HA‐RES‐OPC NPs to the upper medium for 12 h, we observed few nanoparticles in the lower SH‐SY5Y cells, suggesting that the BBB blocks the uptake of nanoparticles. Then, we grafted the B6 peptide into the nanoparticles and repeated the above experiments. We observed that B6‐HA‐RES‐OPC NPs modified with the B6 peptide were approximately four times more able to traverse the mocked BBB (Figure 3c,d). The results illustrate that the B6 peptide can significantly help the nanoparticles to cross the BBB, thus being able to act on the nervous system. For our in vivo test, we used wild‐type c57BL/6J mice injected with B6‐HA‐RES‐OPC NPs through the tail vein at a dose of 1 mg/kg; the control group was injected with the same dose of HA‐RES‐OPC nanoparticles. Twenty‐four hours later, we euthanized the mice and removed some organs for our examination. B6 peptide nanoparticles were significantly deposited in the mouse brain than controls and did not accumulate significantly in the liver, heart, and kidney (Figure 3e,f). Furthermore, the results from the tissue sections showed that the modified nanoparticles remained in the brain at approximately four times the amount of untreated nanoparticles, consistent with the Transwell experiment results (Figure 3g,h). The results show that nanoparticles modified with B6 peptides can cross the BBB to enter the brain tissue, promising to treat neurological diseases.

### 2.4 B6‐RES‐OPC‐HA NPs treatment reduced neuroinflammation and Aβ deposition

**FIGURE 4:**
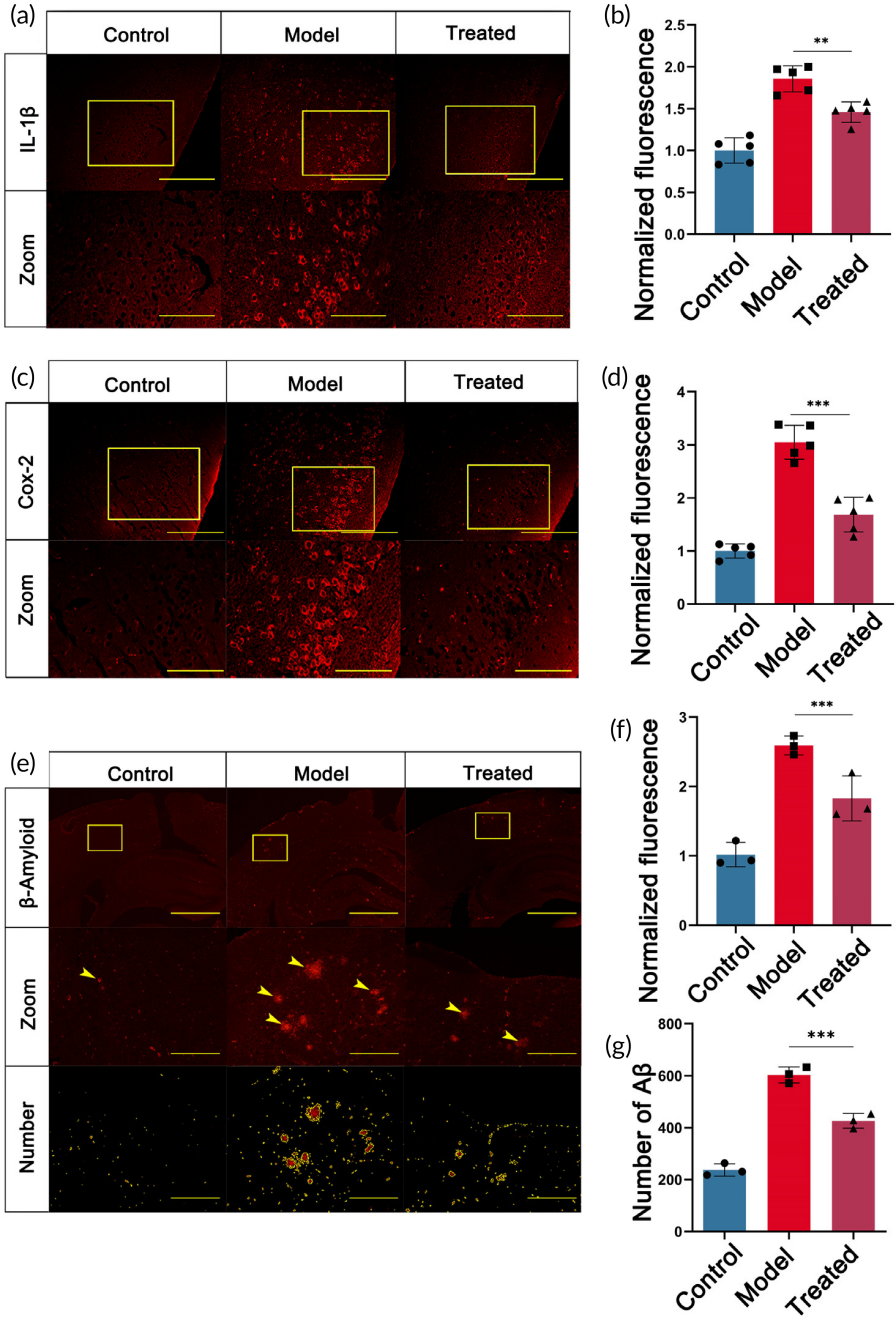
Immunofluorescence of the cerebral cortex of each group of mice after nanoparticle treatment. (a) Immunofluorescence detection and magnification of IL‐1β in various groups of mice, scale bars above are 1 mm and below are 200 μm. (b) IL‐1β fluorescence intensity analysis (*n* = 5). (c) Cortex COX‐2 immunofluorescence detection after nanoparticle treatment in various groups of mice, scale bars above are 1 mm and below are 200 μm. (d) COX‐2 fluorescence intensity analysis (*n* = 5). (e) Immunofluorescence detection of β‐Amyloid in the cortex of each group of mice after nanoparticle treatment, scale bars above are 1 mm; middle are 200 μm, and below are 200 μm. (f) β‐Amyloid fluorescence intensity analysis (*n* = 3). (g) Analysis of the number of β‐amyloid particles. *p < 0.05, **p < 0.01, ***p < 0.001 (*n* = 3).

Inflammation plays a vital role in the AD process, and excessive neuroinflammation causes microglia abnormalities and worsens the disease. Interleukin‐1β (IL‐1β) is an essential factor that regulates cellular inflammation. In the present study, we verified the expression of IL‐1β in the cerebral cortex of experimental mice. For this, B6‐HA‐RES‐OPC NPs were injected at a dose of 1 mg/kg three times a week, and the mice were executed after 4 weeks for examination. Our results demonstrated a significant increase in the number of positive IL‐1β in the brains of AD model mice. However, the amount of positive IL‐1β in the cerebral cortex of treated mice was significantly reduced. This suggests that treatment with nanoparticles significantly improved inflammation occurrence in the central nervous system of mice (Figure 4a,b). Cyclooxygenase‐2 (COX‐2) is a crucial rate‐limiting enzyme associated with inflammation and an important marker in the pathogenesis of AD. We verified that COX‐2 is highly expressed in AD mice in cortical brain areas. However, the COX‐2 in mouse brains was significantly reduced after B6‐HA‐RES‐OPC NPs treatment (Figure 4c,d). It is known that deposition of Aβ is one of AD's most critical pathological features. In the present study, we characterized Aβ deposition in the brains of experimental mice, with the results showing that the accumulation of Aβ in the brains of AD model mice was significantly reduced after the nanoparticle treatment (Figure 4e–g). These results suggest that B6‐RES‐OPC‐HA NPs treatment significantly alleviated neuroinflammation, reduced aβ accumulation, and improved AD‐like behavior in the AD model mice.

### 2.5 B6‐RES‐OPC‐HA NPs improve learning and memory in AD mice

**FIGURE 5:**
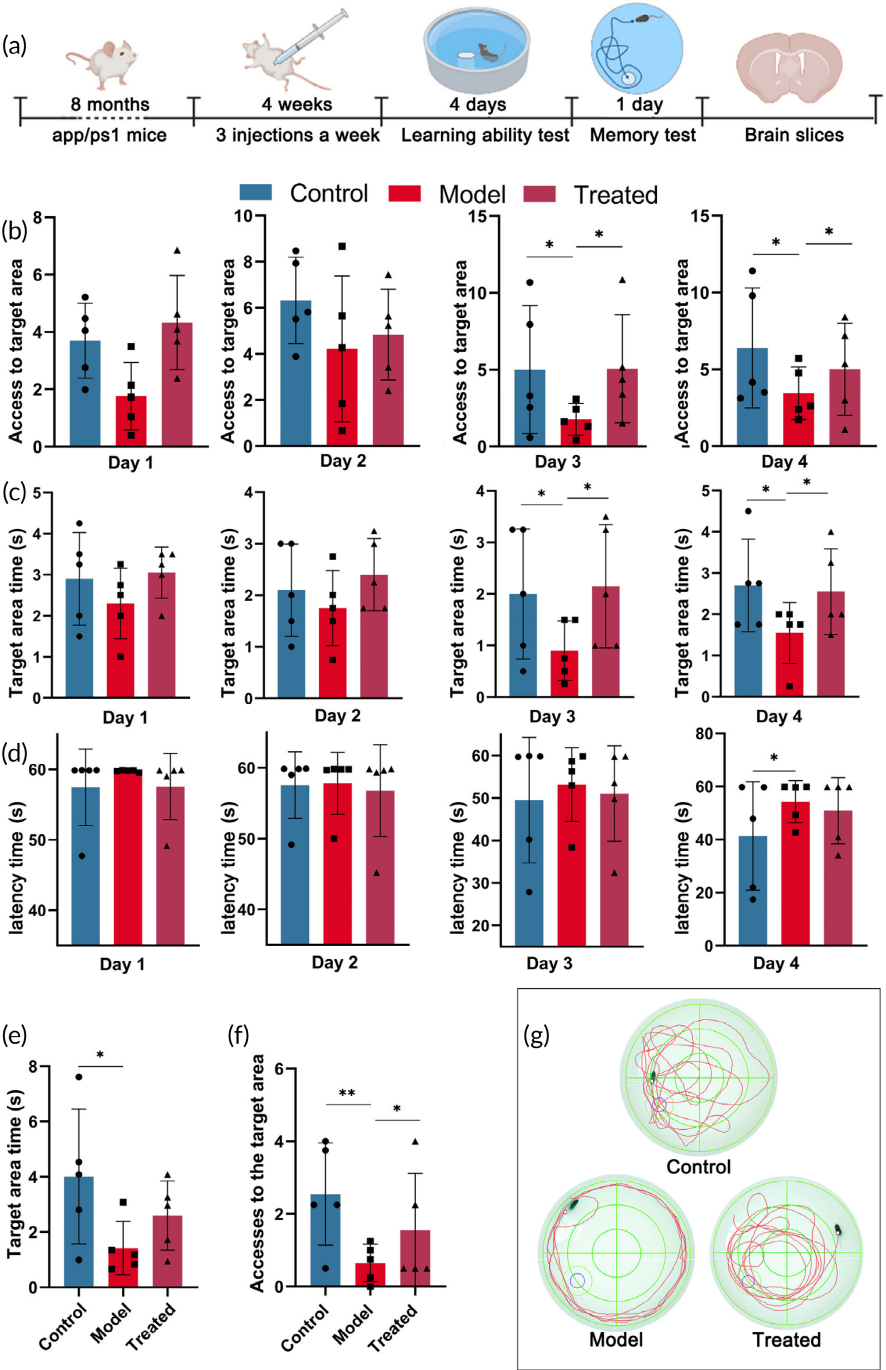
Recovery of learning and memory capacity in AD mice after nanoparticle treatment (*n* = 5). (a) Sketch of the experimental plan. (b) Number of times each group of mice passed through the target area during the learning phase. (c) Time periods spent in the target area by mice during the learning phase. (d) Learning phase latency time. (e) Residence time durations of mice in the target area during memory capacity tests. (f) Number of times mice traversed the target area during the memory capacity test. (g) Trajectories of different groups of mice during memory capacity tests. *p < 0.05, **p < 0.01

To validate the therapeutic effect of B6‐RES‐OPC‐HA NPs on AD, we used APP/PS1 Alzheimer's mice for experiments, where mice were injected with B6‐RES‐OPC‐HA NPs at a dose of 1 mg/kg three times a week for 4 weeks (Figure 5a). After treatment, the morris water maze was used test to examine nanoparticles' effect on learning and memory recovery in AD mice. The mice were divided into wild‐type C57BL/6J mice (control group), APP/PS1 model (model group), and nanoparticle‐treated APP/PS1 model groups (treated group). During the first 4 days of learning, we recorded the time the mice stayed in the target area, the number of times they entered it, and the time needed to find the platform. After 3 days of training, the model group showed significant learning disabilities. However, in the treated group, the duration and number of times the mice entered the target area were significantly higher (Figure 5b,c). At the same time, treated group mice reduced the latency time required to find the platform (Figure 5d). The results indicate that the treatment with nanoparticles significantly improved the learning ability of the AD mice. Afterwards, we assessed the mice's memory capacity on day five. Compared to the model group, the treated group significantly increased their exploration activities in the target area (Figure 5e–g). Taken together, our results have demonstrated that the treatment with B6‐RES‐OPC‐HA NPs can significantly enhance learning and memory abilities in AD mice.

## 3 DISCUSSION

In this study, we constructed homogeneous and stable B6‐HA‐RES‐OPC NPs using HA as the core material. We demonstrated that nanoparticles grafted with two polyphenols, RES and OPC, significantly enhanced the ability to scavenge ROS. Meanwhile, the dual action of HA and polyphenols conferred the anti‐inflammatory ability of the nanoparticles, and the grafting of the B6 peptide gave the nanoparticles a more vital ability to cross the BBB. After treatment, the accumulation of nanoparticles in the mouse brain was apparently observed. Focal inflammatory markers in the brain were significantly reduced, and aβ deposition was reduced by about 30%. Finally, the behavioral experiments demonstrated that the treatment with nanoparticles is able to improve the learning and memory abilities of Alzheimer's mice.

RES and OPC are natural polyphenolic compounds with different antioxidant capacities. It is generally believed that the number and position of phenolic hydroxyl groups determine the antioxidant capacity of polyphenols.^41^ RES has inter‐and para‐phenolic hydroxyl groups and only three in each monomer. However, OPC has up to eight phenolic hydroxyl groups in each monomer and contains all substitution positions.^15^, ^16^ This means that OPC has a more vital ROS scavenging ability in accord with our experimental results. Indeed, the half‐life of polyphenolic compounds in vivo is only 8–14 min, and they are highly susceptible to methylation, sulfation, and loss of reducing ability.^42^ This means that it is crucial to protect polyphenol activity in vivo. In this study, we utilized the carboxyl group in HA to crosslink with the hydroxyl group in polyphenols to form nanoparticles. Results showed that the B6‐HA‐RES‐OPC NPs protected the activity of polyphenols in vivo. Notably, our previous study has demonstrated that HA inhibits neuroinflammation and can reduce ROS production at the source by protecting mitochondria.^29^ B6‐HA‐RES‐OPC NPs significantly reduced neuroinflammation and decreased Aβ deposition in vivo, suggesting that B6‐HA‐RES‐OPC NPs, formed from two polyphenols, can synergically and enhance the ROS scavenging ability.

Previous studies have achieved nanoparticle accumulation in the brain using HA‐based grafting of RES with curcumin via intranasal administration.^43^ However, the absence of a BBB crossing capability limits the delivery of nanoparticles to the target sites.^44^ BMEC co‐culture with astrocytes is an effective BBB mimicking system.^45^ By this system, our results show that the ability of nanoparticles to cross the BBB is enhanced fourfold by grafting B6 peptides. It should be noted that the BBB system is an in vitro constructed system, and its functions may not be the same as the native one. However, animal experiments showed that grafting of B6 peptides significantly increased the accumulation of nanoparticles in the mouse brain. On this basis, we can reasonably speculate that the grafted B6 peptide can enhance nanoparticles' ability to cross the BBB, thus improving nanoparticle bioavailability.

It is noted that a number of AD models have been reported in the literature, but none of the single models can fully mimic all AD features.^46^ The model of APP/PS1 double transgenic mice employed in the present study has been commonly used.^47^ Some limitations are associated, such as the one that no extensive neuronal loss occurs in this model. Also, this model completely fails to simulate tau protein abnormalities during AD. However, APP/PS1 transgenic mice develop the amyloid plaques in an age‐dependent manner and show the associated memory deficits by the Morris water maze test. APP/PS1 transgenic mice faithfully mimic most AD symptoms for disease studies.^48^ More importantly, the APP/PS1 model can faithfully model the neuroinflammation and ROS abnormalities of early AD, which is essential for testing the nanoparticles. Therefore, we chose this model in preference. Notably, tau protein deposition is a major hallmark of AD^48^; tau dysfunction contributes to AD in a complex manner.^49^ Given that the APP/PS1 model lacks tau abnormalities, studies on the B6‐RES‐OPC‐HA NPs as applied to the tau proteins and related models would be urged in the future.

Our results show that B6‐RES‐OPC‐HA NPs achieved scavenging of ROS at lower concentrations while significantly attenuating LPS‐induced inflammatory activation in microglia. Indeed, neuroinflammation and thus, nerve damage is an essential mechanism for AD development.^50^ Studies showed that neuroinflammation is an equally important AD marker event as Aβ deposition.^51^ Polyphenols can mitigate the associated neuroinflammatory events by scavenging ROS.^11^ Intriguingly, HA has an excellent anti‐inflammatory effect. Also, studies have demonstrated the good neuroinflammatory inhibitory effect of HA nanoparticles, thus improving cognitive impairment in mice.^20^, ^52^ IL‐1β plays a central role in the initiation and regulation of neuroinflammation and is the most significantly elevated inflammatory factor in neuroinflammation.^53^ Also, COX‐2 has been reported to be an essential indicator for diagnosing AD.^54^ Our results show that IL‐1β and COX‐2 significantly decrease in the cerebral cortex of AD mice treated with B6‐RES‐OPC‐HA NPs, consistent with the results from the in vitro experiments. It is noted that both polyphenols and HA have anti‐inflammatory effects. In the present study, we did not distinguish their roles in this regard, which will be a focus in subsequent studies.

In the course of AD, Aβ deposition is the dominant diagnostic criterion for AD.^55^ Our results show that AD mice treated with nanoparticles significantly reduced Aβ in the cerebral cortex, indicating a good Aβ clearance effect. However, studies have shown that polyphenols can act directly on Aβ to reduce aggregation.^56^ Meanwhile, other studies have indicated that polyphenols reduce Aβ by scavenging ROS to reduce neuroinflammation.^57^ We hypothesize that our nanoparticles exploit both properties of polyphenols, along with the anti‐inflammatory capacity of HA, to achieve Aβ clearance in the brain. Further studies are urged to investigate the mechanism by which nanoparticles reduce Aβ.

In summary, we used two polyphenols, RES and OPC, to crosslink with HA to form stable, homogeneous nanoparticles, and enhanced the BBB crossing ability by grafting them with B6 peptides. The nanoparticles showed significant ROS scavenging and anti‐inflammatory effects. The treatment with B6‐HA‐RES‐OPC nanoparticles significantly reduced Aβ deposition in the brains of AD mice, reduced neuroinflammation and improved learning and memory abilities. Taken together, the use of the B6‐HA‐RES‐OPC nanoparticles would represent a promising strategy for treating AD.

## 4 MATERIALS AND METHODS

### 4.1 Animal studies

Five‐month‐old male APPswe/PS1dE9 (App/PS1) double transgenic mice (APPswe/ PS1dE9 transgenic mice expressing chimeric mouse/human APP695 and harboring the Swedish K670M/N671L mutations and human PS1 with exon 9 deletion) and Five‐month‐old male C57BL/6J mice were used for in vivo experiments. All the mice were maintained at a controlled temperature and humidity on a 12‐h light/dark cycle (lights on at 7:00) and provided regular rodent chow and sterilized tap water ad libitum. All mouse experiments were approved and performed according to the guidelines of the Harbin Institute of Technology Committee on Animal Resources (IACUC‐2022054).

In animal experiments, the control group was wild‐type C57BL/6J mice injected with saline according to body weight; the model group was AD model mice injected with saline according to body weight, and the treated group was AD mice injected with B6‐HA‐RES‐OPC NPs according to body weight. Each animal is one unit.

### 4.2 Cell and antibody

SH‐SY5Y, BV2, BMECs, and C8‐D1A(Astrocyte type I clone) cells were from the American Type Culture Collection (ATCC; Manassas, VA, USA) the chemicals from Sigma Aldrich Co. (St. Louis, MO, USA) cell culture reagents from ThermoFisher Scientific (Waltham, MA, USA) anti‐beta‐Amyloid and Anti‐PTGS2(COX‐2) Antibodies from Boster Biological Technology Co. Ltd. (Pleasanton, CA, USA), and anti‐IL‐1 beta Antibody from Abcam plc (Cambridge, UK).

### 4.3 Cell Culture

BV2 and C8‐D1A cells were maintained in DMEM with 10% (v/v) FBS. BMECs cells were maintained in DMEM: F12 with 10% (v/v) FBS. SH‐SY5Y cells were maintained in 43.5% (v/v) MEM with 43.5% (v/v) F12, 10% (v/v) FBS, 1% (v/v) Gluta‐max, 1% (v/v) sodium pyruvate, and 1% (v/v) NEAA. The cell culture conditions were set as 5% CO_2_ and 37°C in a cell incubator with a humidified atmosphere, and the cells were subcultured when reaching 80% confluency.

### 4.4 Synthesis of HA‐RES‐OPC NPs

A mixture was prepared from 19 ml of formamide (1.0 mg/ml) and dimethylformamide (0.9 mg/ml) with 0.3 g of HA, followed by adding 0.1 g of toluenesulfonic acid and stirring for 1 h at 50°C. The procedure was to thoroughly dissolve 0.1 g of RES and 0.1 g of OPC and add an appropriate amount of DCC/DMAP, which were then set for 24 h at room temperature in a dark room. After that, the procedure was to add 40 ml of ethanol, shake vigorously, and centrifuge (15,000 g 15 min). Eventually, the precipitate or particles were taken out and washed twice with ethanol, then stored in a dialysis bag(3500 KDa) for 48 h, followed by lyophilization. The samples of particles were then stored at −20°C for the subsequent experiment.

### 4.5 Grafting of B6 peptide

HA solution (400 mg, 1 mmol) was dissolved in 40 ml MES buffer (0.1 M MES, 0.3 M NaCl, and pH 6.5) at a concentration of 10 mg/ml. To this reaction mixture, 1‐ethyl‐3‐(3‐dimethylamino propyl) carbodiimide (EDC; 2 mmol) and *N*‐hydroxysuccinimide (NHS; 2 mmol) were added and stirred at room temperature for 10 min. A solution of B6 peptide (CGHKAKGPRK; 0.5 mg/ml, 100 μl) and HA‐RES‐OPC NPs (1.25 mg/ml, 1 ml) was stirred at room temperature for 24 h. The procedure was to add 40 ml of ethanol, shake vigorously, centrifuge (15,000 g 15 min), then store in dialysis bag (3500 KDa) for 72 h, followed by lyophilization. The obtained peptide was stored at −20°C for the subsequent experiment.

### 4.6 Characterization of the nanoparticles

Dynamic light scattering (DLS; Brookhaven) was used to characterize the average hydrodynamic diameter of the NPs in an aqueous solution. A 30 mW argon‐ion laser was used for DLS measurements at an angle of 90° at room temperature. Then, 3 ml of NP solution was added to the colorimetric dish and used for measurement after standing for about 1–2 min. The particle size and its distribution were subsequently detected. After that, 1 ml of NP solution was taken from the colorimetric dish to detect the Zeta potential on the surface of NPs. Ten measurements were carried out per cycle and repeated six times.

### 4.7 Morphological observation of NPs by scanning electron microscopy

NPs were monodispersed onto the copper slides and air‐dried, then coating a thin layer of gold using an Agar HR sputter coater. The specimen was then imaged using field emission scanning electron microscopy (HITACHI S4300‐SEM) at 5 kV acceleration voltage and 12 μA beam current. Six images were captured from each sample. The size of the NPs was measured using LSM Image Browser software; a total of 50 mg NPs were analyzed.

### 4.8 DPPH free radical scavenging capacity assay

The DPPH (0.1 mmol/L) solution was prepared using 95% ethanol. The solution of 100 μl to be measured was mixed with 150 μl of DPPH solution, and the same volume of PBS was used for the control group to mix with the DPPH solution, and the reaction was carried out at room temperature and protected from light for 30 min. After that, the absorbance of the solution was measured at 517 nm by a microplate reader (Molecular Devices, San Jose, CA, USA).

### 4.9 Characterization of the ROS scavenging capacity of nanoparticles

The ability of nanoparticles to protect SH‐SY5Y cells after ROS stimulation was measured using the reactive oxygen species assay kit (Beyotime Biotechnology; Shanghai, China). After SH‐SY5Y cells were walled, the corresponding different concentrations of nanoparticles were added and incubated overnight. The oxidative stress of the cells was increased by adding ROSUP reagent from the kit. After 4 h, the cells were washed three times using a serum‐free medium, and the cell surface was covered with ROS fluorescent probes. After 10 min, the cells were washed three times using a serum‐free medium. Cell fluorescence was recorded using a fluorescence microscope, and the area of the fluorescent area was counted using Image J.

### 4.10 Detection of the anti‐inflammatory ability of nanoparticles

The protective effect of nanoparticles on BV2 cells after LPS stimulation was tested using the nitric oxide assay kit (Beyotime Biotechnology; Shanghai, China). First, a standard curve between NO concentration and absorbance was established. After the cells were plastered and LPS was added to make the final concentration of 1 μM, different concentrations of antioxidant materials were added separately. After overnight, a small amount of culture medium supernatant was collected daily, and its absorbance at 540 nm was tested after treatment with reagents in the kit. The concentration of NO in each sample was calculated from the standard curve.

### 4.11 Nanoparticle BBB crossing assay

The transwell (Corning, NY, USA) was placed on a 24‐well plate, and the upper layer was cultured overnight with a 1:2 ratio of mixed BMECs and C8‐D1A cells. Once the vascular endothelial cells had completely covered the bottom layer of the transwell under the microscope, coumarin 6‐labeled nanoparticles (0.01 mg/ml) were added dropwise to the upper chamber. Replace the lower layer with a 24‐well plate where SH‐SY5Y cells are cultured. Periodically photograph the fluorescently labeled nanoparticles in the lower layer of SH‐SY5Y cells using a fluorescence microscope and count the area of the fluorescent area using Image J.

### 4.12 Morris water maze test

We performed the MWM test described previously with slight modifications.^58^ The device consists of a circular pool with a diameter of 122 cm filled with water (22–24°C). A nontoxic coating was added to the water to make it opaque. An escape platform with a diameter of 10 cm was placed one foot below the water surface. We divided the experimental pool into four equal quadrants A–D. During the first 7 days, the mice adapt to their environment with the experimental staff. In the following four experiments, tests were conducted at 10 am daily, and mice were placed in a maze 12.5 cm from the edge of the pool in a randomized sequence. Each trial lasted 60 s, and we recorded the time taken to find the hidden platform (latency). Test trials were performed 24 h after the last training trial, during which the underwater platform was removed. Mice were randomly grouped before the behavioral experiment, and the operator was blinded during the experiment. We counted all data without any exclusions. All training and probe test trials were recorded using Panlab Smart 3.0 software.

### 4.13 Animal in vivo imaging

The mice were euthanized by spinal dislocation after deep anesthesia with isoflurane as per the relevant small animal welfare policy. After the confirmation of mouse death, imaging was performed using an in vivo imaging system (IVIS Spectrum; PerkinElmer), and the content of nanoparticles in each organ was analyzed.

### 4.14 Immunostaining

The mice were perfused with paraformaldehyde (4%) under deep anesthesia, and the brains were postfixed in 4% paraformaldehyde overnight. A freezing microtome (Leica) was employed to section the brains into 40 μm segments. The sections were permeabilized and blocked at room temperature for 1 j using PBS consisting of Triton X‐100 (0.2%) and normal goat serum (10%). The brain sections were incubated overnight with the primary antibody in PBS consisting of Triton X‐100 (0.2%) and normal donkey serum (10%) at 4°C. The primary antibodies were visualized using conjugated secondary antibodies using Alexa Fluor 594 (Abcam). Cell nuclei were visualized using DAPI (1:1000). A confocal microscope (LSM 880; Zeiss) was employed to acquire images under similar settings for all the test conditions. We utilized two coronal sections per mouse for each test condition from a specified number of mice and used ImageJ to analyze the images.

### 4.15 Statistical methods

All data are shown as the mean ± standard deviation (SD). The means of the two groups were compared using a two‐tailed Student's *t*‐test; Welch's correction was used in case of unequal variances. The means of the three groups were compared with a one‐way analysis of variance (ANOVA) with Tukey's multiple comparison post hoc test. All analyses were performed using GraphPad Prism 8 (GraphPad Software, San Diego, CA, USA). The value of *p* < 0.05 was accepted as statistically significant.

## AUTHOR CONTRIBUTIONS

**Bosong Zhang:** Conceptualization (equal); data curation (equal); formal analysis (equal); investigation (equal); methodology (equal); resources (equal); software (lead); validation (equal); visualization (lead); writing – original draft (lead). **Yufang Zhao:** Conceptualization (equal); formal analysis (equal); investigation (equal); methodology (equal); supervision (equal); validation (equal); writing – review and editing (equal). **Kai Guo:** Investigation (equal); methodology (equal); validation (equal). **Hui Tian:** Investigation (equal); methodology (equal); validation (equal). **Cao Wang:** Investigation (equal); methodology (equal); validation (equal). **Ruiqi Wang:** Investigation (equal); methodology (equal). **Yue Chen:** Investigation (equal); methodology (equal); validation (equal). **Xiongbiao Chen:** Supervision (equal); writing – review and editing (equal). **Hongxia Zheng:** Writing – review and editing (equal). **Bingxin Gao:** Investigation (equal); methodology (equal); validation (equal). **Jieyi Shen:** Investigation (equal); methodology (equal); validation (equal). **Weiming Tian:** Conceptualization (equal); data curation (equal); formal analysis (equal); funding acquisition (lead); investigation (equal); methodology (equal); project administration (equal); supervision (equal); writing – review and editing (equal).

## CONFLICT OF INTEREST

There are no conflicts to declare.

### PEER REVIEW

The peer review history for this article is available at https://publons.com/publon/10.1002/btm2.10459.

## Supporting information


**Figure S1.** The chemical process of nanoparticle construction. (a) Acidification process of HA to obtain activated carboxyl groups. (b) Hydroxyl groups in RES and OPC are used to crosslink with the carboxyl groups of HA to form nanoparticles and protect the phenolic hydroxyl groups to maintain the antioxidant capacity of the nanoparticles.
**Figure S2.** Complementary to nanoparticle characterization. (a) Macroscopic morphology of nanoparticles dispersed in the aqueous phase. (b) Nuclear magnetic resonance verifies the successful construction of nanoparticles.
**Figure S3.** HE staining of the major organs of mice after B6‐HA‐RES‐OPC NPs treatment. There was no significant damage to the mice's heart, liver, spleen, and kidneys, scale bars are 200 μm.

## Data Availability

The data that support the findings of this study are available from the corresponding author upon reasonable request.
