# Correction to “Macromolecular nanoparticles to attenuate both reactive oxygen species and inflammatory damage for treating Alzheimer's disease”

**DOI:** 10.1002/btm2.70052

**Published:** 2025-08-01

Zhang B, Zhao Y, Guo K, et al. Macromolecular nanoparticles to attenuate both reactive oxygen species and inflammatory damage for treating Alzheimer's disease. *Bioeng Transl Med*. 2023;8(3):e10459.

In the DAPI channel of figure 3g. The pictures above and below have been reversed. This is incorrect. The correct pictures should be reordered.

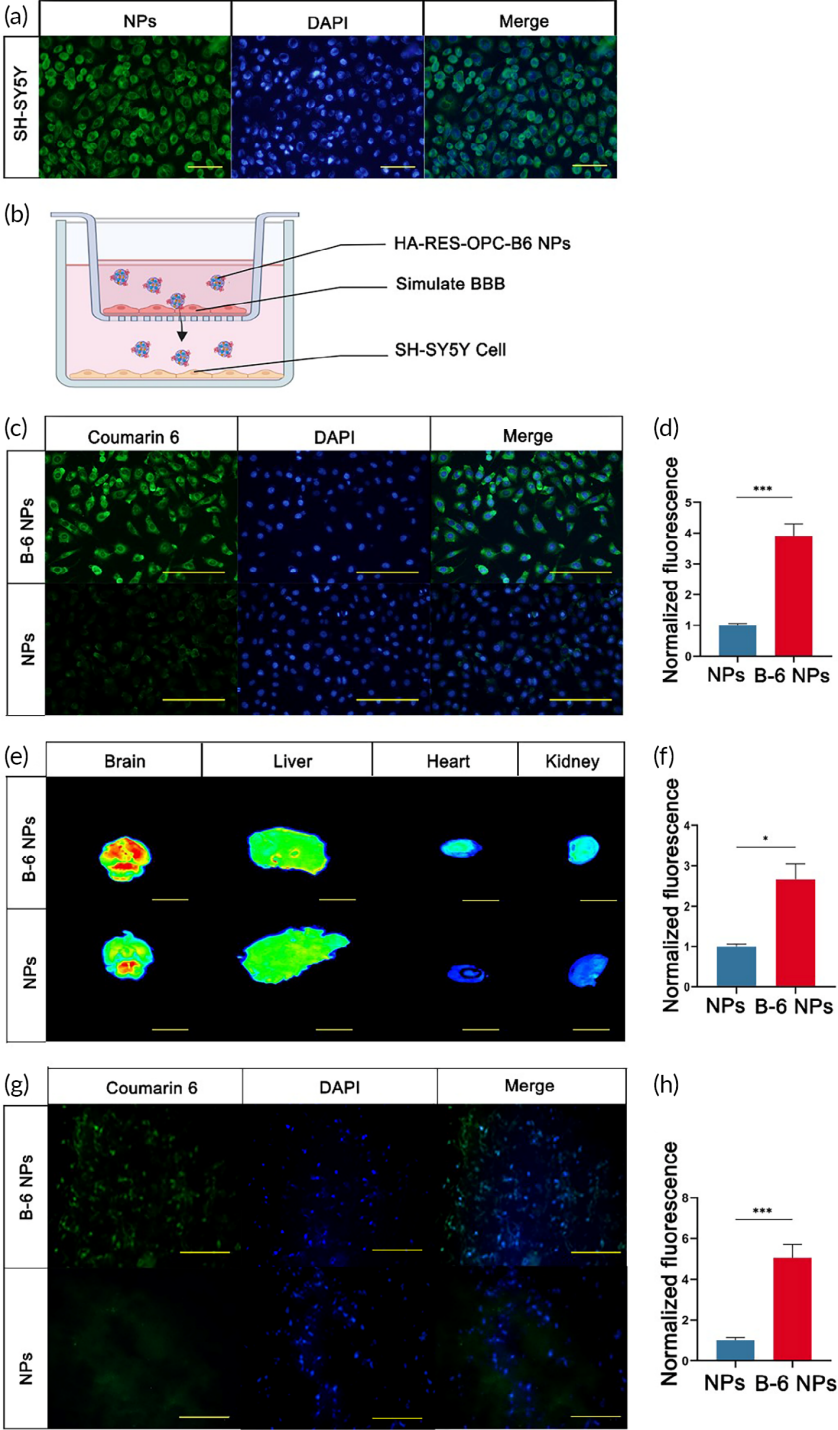

We apologize for this error.

