# Supplementary material for: Macromolecular nanoparticles to attenuate both reactive oxygen species and inflammatory damage for treating Alzheimer's disease
Source: Bioeng Transl Med. 2022 Nov 29;8(3):e10459. doi: 10.1002/btm2.10459 (PMC10189435; doi:10.1002/btm2.10459)
Supplement: Supplementary file 1 — Figure S1. The chemical process of nanoparticle construction. (a) Acidification process of HA to obtain activated carboxyl groups. (b) Hydroxyl groups in RES and OPC are used to crosslink with the carboxyl groups of HA to form nanoparticles and protect the phenolic hydroxyl groups to maintain the antioxidant capacity of the nanoparticles. Figure S2. Complementary to nanoparticle characterization. (a) Macroscopic morphology of nanoparticles dispersed in the aqueous phase. (b) Nuclear magnetic resonance verifies the successful construction of nanoparticles. Figure S3. HE staining of the major organs of mice after B6‐HA‐RES‐OPC NPs treatment. There was no significant damage to the mice's heart, liver, spleen, and kidneys, scale bars are 200 μm. [file BTM2-8-e10459-s001.docx]

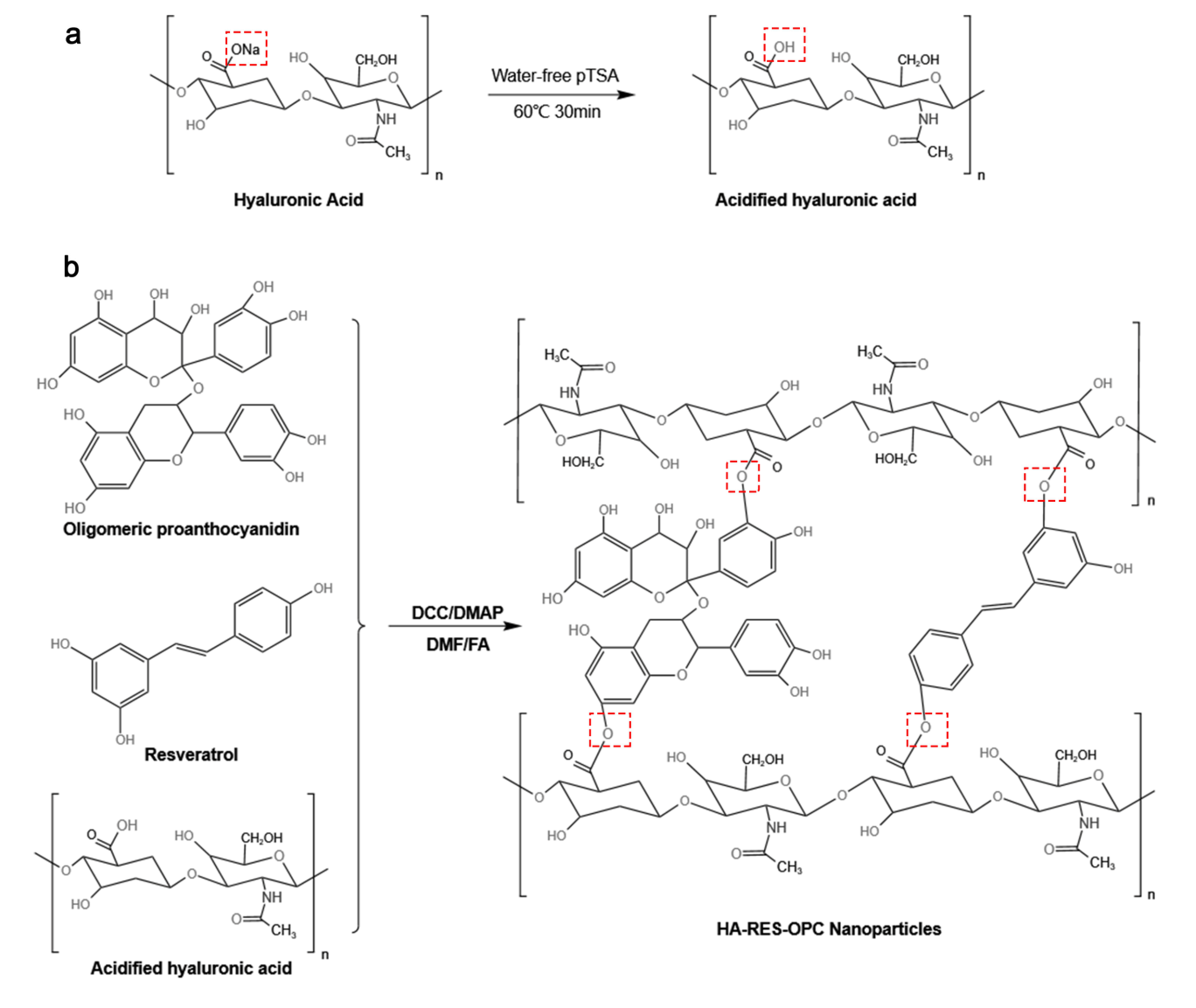
**Figure S1.** **The chemical process of nanoparticle construction. (a)** Acidification process of HA to obtain activated carboxyl groups. **(b)** Hydroxyl groups in RES and OPC are used to crosslink with the carboxyl groups of HA to form nanoparticles and protect the phenolic hydroxyl groups to maintain the antioxidant capacity of the nanoparticles.


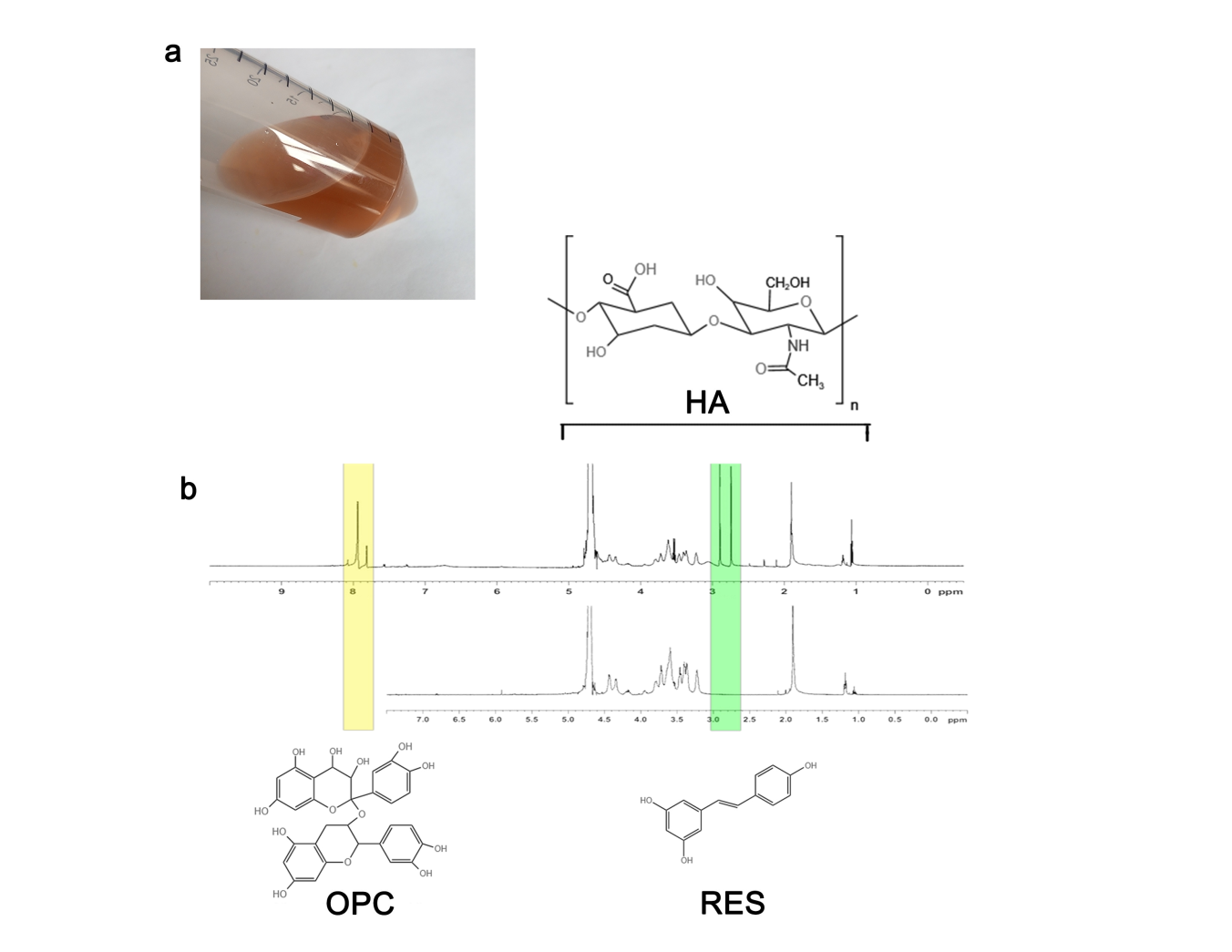
**Figure S2.** **Complementary to nanoparticle characterization** **(a)** Macroscopic morphology of nanoparticles dispersed in the aqueous phase. **(b)** Nuclear magnetic resonance verifies the successful construction of nanoparticles.


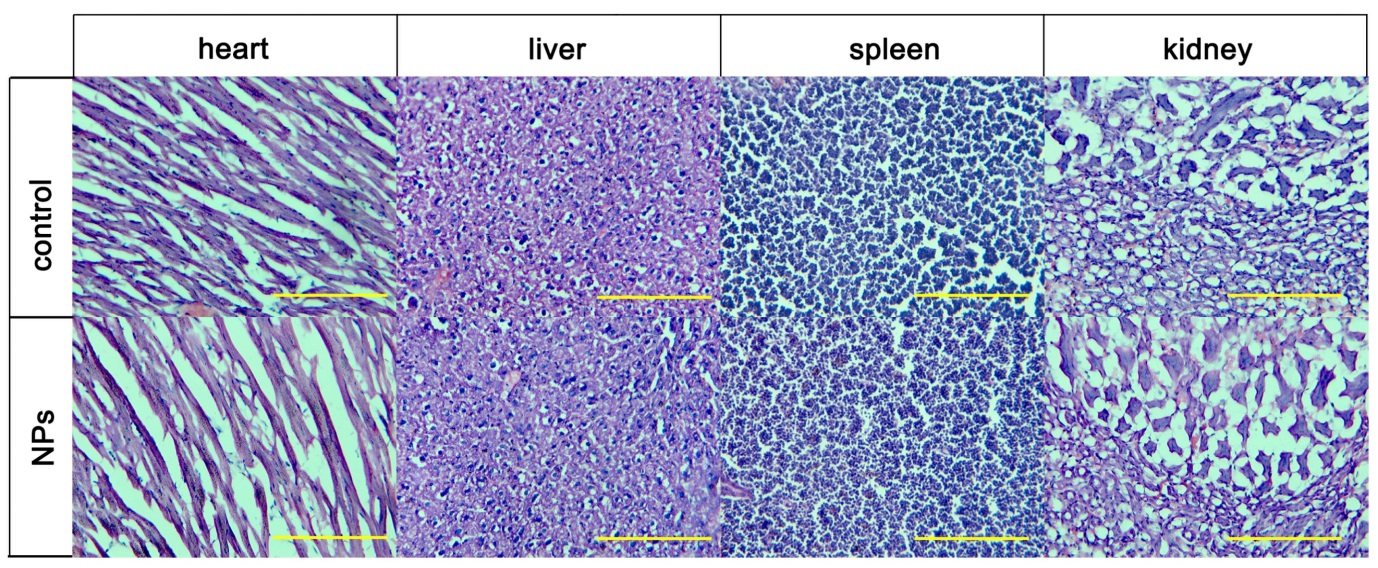
**Figure S3.** HE staining of the major organs of mice after B6-HA-RES-OPC NPs treatment. There was no significant damage to the mice's heart, liver, spleen and kidneys, scale bars are 200μm.
